# Assessment of Biochemical Parameters in 8- and 9-Year-Old Children with Excessive Body Weight Participating in a Year-Long Intervention Program

**DOI:** 10.3390/jcm12206560

**Published:** 2023-10-16

**Authors:** Dominika Raducha, Joanna Ratajczak, Ewa Kostrzeba, Ewa Berus, Mieczysław Walczak, Elżbieta Petriczko

**Affiliations:** 1 Department of Paediatrics, Endocrinology, Diabetology, Metabolic Diseases and Cardiology of Developmental Age, Pomeranian Medical University in Szczecin, 71-252 Szczecin, Poland; dominikareszke@gmail.com (D.R.); ewa.berus@pum.edu.pl (E.B.); mieczyslaw.walczak@pum.edu.pl (M.W.); elzbieta.petriczko@pum.edu.pl (E.P.); 2 Institute of Physical Culture Sciences, University of Szczecin, 70-237 Szczecin, Poland; joannasza@poczta.onet.pl

**Keywords:** childhood obesity, cardiometabolic risk, intervention program

## Abstract

Childhood obesity represents a significant challenge both clinically and socio-economically. This study aimed to assess specific biochemical parameters, particularly glucose, insulin and lipid profile, before and after a year-long intervention program in 8- and 9-year-old children with excessive body weight living in Szczecin, Poland from 2016 to 2018. The research comprised two phases: screening in elementary schools and intervention in the outpatient clinic of the clinical Pomeranian Medical University hospital. Out of 11,494 8- to 9-year-olds in Szczecin, 42.54% (4890) participated in the screening. In the intervention phase, 515 children were examined. Anthropometric measurements were recorded at each visit, and blood samples were collected during the first and fourth visits. In the statistical analysis, the Kolmogorov–Smirnov, t-Student and ANOVA tests were employed (with statistical significance when *p* ≤ 0.05). Results highlighted a significant proportion of children exhibiting disruptions in carbohydrate and lipid metabolism. A total of 8.6% of participants had elevated total cholesterol, 9.7% had reduced HDL, 13.4% had elevated LDL, and 21.2% had elevated triglycerides. Initially, abnormal fasting glucose was detected in 4.7% of children, and elevated insulin levels in 3.1%. Metabolic disorders persisted post-intervention despite BMI improvement. The results emphasize the necessity for prolonged programs with frequent follow-ups targeting weight normalization in children.

## 1. Introduction

The results of the studies confirm that childhood obesity remains one of the most significant challenges in 21st-century medicine. The troubling increase in the number of children with excessive body weight is concerning. In 2020, excess body weight affected 39 million children under the age of 4. Despite the ongoing serious and unresolved issues of hunger and underweight, the majority of the global population resides in countries where deaths due to excessive body weight are more prevalent than due to underweight (with exceptions in parts of Sub-Saharan Africa and Asia) [1]. Childhood obesity substantially elevates the risk of complications that impact both the quality and length of life. One of the more serious complications secondary to excessive body weight is metabolic disturbances, which can manifest as insulin resistance, impaired glucose tolerance, or type 2 diabetes. Their occurrence depends on the severity of obesity, the duration of the condition, visceral fat mass, and family history of diabetes. In obese children, the most common primary cause of carbohydrate metabolism disorders is peripheral tissue resistance to insulin action [2,3]. Currently, the greatest influence on the development of insulin resistance in adolescents is exerted by obesity.

There is a general consensus that insulin resistance predisposes the development of glucose metabolism disorders, dyslipidemia, and high blood pressure, which are components of metabolic syndrome. This, in turn, is a later factor in the development of type 2 diabetes, cardiovascular diseases, and many others, and can also serve as an independent predictor of cardiovascular risk [2]. The prevalence of type 2 diabetes among children and adolescents has recently rapidly increased worldwide, which is correlated with the global rise in obesity rates among the youngest patients [4,5]. Children with obesity are 4-fold more likely to develop type 2 diabetes compared to children with a normal BMI [6]. Thirty years ago, type 2 diabetes was considered a rare disease in the pediatric population. Up until the mid-1990s, only 1–2% of children with diabetes were classified as having type 2 diabetes. However, with the increase in obesity, the frequency of type 2 diabetes has risen depending on the studied population, ranging from 8 to 45% of all new diabetes cases reported in children and adolescents [7]. A study conducted by Starzyk et al. revealed that DM2 accounts for 10% of all diagnosed cases of diabetes in Caucasian youth. Additionally, prediabetic conditions such as impaired glucose tolerance were diagnosed in 10–27% of obese children. The average age of DM2 diagnosis in youth was 13 years [8,9]. On the other hand, a comprehensive meta-analysis conducted by Cioana revealed that 75.27% of children with type 2 diabetes had obesity, and 77.24% were obese at diagnosis [10].

In recent years, the causes of lipid disorders in children have also changed due to the steadily growing percentage of children with excessive body weight. Genetic disorders used to be the most common cause of dyslipidemia. In recent decades, an increase in dyslipidemia secondary to obesity has been observed [11]. Most constitutional lipid disorders occur independently of excessive body weight, yet obesity can facilitate the expression of certain lipid disorders. In a Polish study examining lipid disorders published in 2020 performed on a group of 1948 children and adolescents aged 6–15 with excess body weight, at least one lipid disorder was described in 38.23% of girls and 40.51% of boys with overweight and obesity. The most prevalent lipid disorders were decreased high-density lipoprotein cholesterol (HDL-C) levels, found in 20.55% of girls and 23.79% of boys, and elevated low-density lipoprotein cholesterol (LDL-C), observed in 15.31% of girls and 14.25% of boys. There was no strong association between lipid disorders and age, sex, birth weight, gestational age at birth, or body composition [12]. Moreover, cardiovascular risk indicators such as reduced HDL cholesterol fraction, high systolic and diastolic blood pressure, and elevated triglyceride levels increased with the severity of obesity [13]. The rise in the frequency of excessive body weight and the large scale of this phenomenon have led to more attention being paid to coexisting diseases and complications resulting from obesity. The degree of obesity in children and adolescents has significant clinical implications. Some of them once considered long term and characteristic of adult obesity are now being increasingly diagnosed in children and adolescents. Therefore, the role of preventive programs funded by public resources is crucial.

The objective of this study was not only to assess the biochemical parameters before and after the completion of a year-long intervention activities in 8- and 9-year-old children with excessive body weight but also to collect epidemiological data on the prevalence of overweight and obesity among this study population and to determine the effectiveness of medical intervention in the form of a year-long interdisciplinary collaboration. Studies assessing the prevalence of excess body weight in children living in Szczecin and evaluating the risk of obesity-related complications have not been conducted so far. The choice of the research topic stemmed from the need for epidemiological studies in this region providing information about the scale of the excessive bodyweight prevalence in children. The other aim of this study was to promote a healthy lifestyle and counteract the development of lifestyle diseases.

Given the insufficient level of knowledge in society regarding the causes and complications of excess body weight, the program had a significant societal character by introducing health-promoting measures and improving the awareness of the entire patient environment, especially their families and schools. Cooperation with educational institutions was crucial to ensure that preventive and intervention measures were as effective as possible. Importantly, this study had a societal nature and was directed at a large percentage of recipients to provide real assistance for as many children as possible. Furthermore, this research aimed to demonstrate how to formulate preventive and intervention programs to ensure their long-term effectiveness at the highest level.

## 2. Materials and Methods

### 2.1. Study Design

This study had a prospective nature. The study group consisted of children enrolled in the “Courageous Eight” program aiming to reduce the body weight of school-aged children living in Szczecin, Poland. The program was carried out by the Independent Public Clinical Hospital No. 1 of the Pomeranian Medical University in Szczecin, the University of Szczecin, and the Non-Public Health Care Institution “School Medicine” (“SZKOLMED”) in Szczecin.

This study consisted of two main stages: Stage I—screening, Stage II—intervention. The program was implemented over three consecutive years: 2016, 2017, and 2018. In 2016, children born in 2008 were examined; in 2017, children born in both 2008 and 2009; while in 2018, those born in 2010. The entire program was financed by the Municipality of Szczecin and was developed under the license of the Polish Society of Health Programs in Gdańsk, receiving its positive evaluation. The Society also acted as an auditor of the project.

#### 2.1.1. First Stage of the Program

The initial phase of the program involved screening and a comprehensive health analysis. All eight- and nine-year-old children in Szczecin (those attending second and third grades) whose parents provided consent for participation in the program were examined. The screenings were conducted in all primary schools in Szczecin, including public, private, and special schools. A qualified and additionally trained team of school nurses carried out the assessments. They diligently input all patient data and test results into a computer program specifically developed for this project.

During the first phase, the examinations involved measurements of basic anthropometric parameters, such as body height, body weight, BMI, waist and hip circumference. Other assessments included arterial blood pressure, body composition analysis, and cardiorespiratory fitness (physical endurance). BMI (Body Mass Index) and WHR (Waist-to-Hip Ratio) were calculated. Children with excessive body weight, identified by a BMI ≥ 90th percentile, proceeded to the next phase of the program. The obtained results were compared against Polish growth charts developed from OLAF and OLA studies conducted between 2007 and 2012 [14]. The criteria established by CDC were adopted: BMI ≥ 85th–95th percentile for overweight and BMI ≥ 95th percentile for obesity [15].

#### 2.1.2. Second Stage of the Program

The second phase of the program involved medical intervention, conducted in an outpatient specialist clinic. The qualification criterion was BMI ≥ 90th percentile concerning the norm for gender and age, in reference to the growth charts for the Polish population (OLAF and OLA) [14]. Children qualified for the second stage remained under interdisciplinary care for one year. During this period, they participated in four specialized visits. The planned interval between the 1st and 2nd visits was 3 months, 3 months between the 2nd and 3rd visits, and 6 months between the 3rd and 4th visits. During each of these meetings, the child underwent four appointments with a physician, dietitian, physical activity specialist, and psychologist. Throughout the one-year program, each participant was expected to undergo 16 specialized consultations in the presence of at least one legal guardian. Whole families were welcome, especially members of the family directly responsible for the child’s diet. Patients who decreased their BMI percentile to <90 according to the OLAF and OLA growth charts were eligible to conclude their participation in the program. However, some patients decided to continue the program despite reaching the BMI centile allowing its completion.

Anthropometric parameters, arterial blood pressure, body composition analysis, and cardiorespiratory fitness (physical endurance) were reassessed. Additionally, biochemical parameters taken during the first and fourth visits were evaluated to assess carbohydrate and lipid metabolism. Moreover, to promote an active lifestyle and alternative ways of spending leisure time, various sports activities were organized: swimming pool sessions, forest adventure hikes, soccer training with players from the local sports club “Pogoń”, general developmental exercises, and family bike trips. Recognizing that the foundation of lifestyle change lies in the support of parents and legal guardians, educational workshops lasting several hours were prepared specifically for this audience. These workshops were conducted by a physician, dietitian, psychologist, and physical activity specialist. Additionally, a series of educational training sessions were provided for teachers and school cafeteria staff.

During each visit, patients received individual recommendations as well as referrals for laboratory tests, aimed at assessing the causes and potential complications of excessive body weight. Throughout the program, laboratory tests were conducted twice:

- Within 7 days after the first visit, fasting glucose, insulin concentrations, lipid profile (total cholesterol, LDL and HDL cholesterol fractions, and triglycerides), ALT, AST, TSH, and fT4 were measured.
- Within 7 days before the fourth visit, fasting glucose, insulin concentrations, and the lipid profile (total cholesterol, LDL and HDL cholesterol fractions, and triglycerides) were assessed to gauge changes.

Not every child who underwent the first visit could be evaluated for all biochemical parameters. Some finished their participation due to program success (reduction in BMI centile), while others discontinued or did not undergo tests due to child anxiety related to blood collection. Concentrations of AST, ALT, fT4, and TSH were assessed in a small subset of children during the fourth visit. The special focus was placed on tests describing carbohydrate and lipid metabolism disturbances. In case of abnormalities in laboratory test results, specific recommendations were provided to patients, including follow-up with a primary care physician or timely consultation with an outpatient endocrinology clinic to expand diagnostic evaluation.

### 2.2. Study Population

#### Projected and Actual Number of Participants

**Figure 1:**
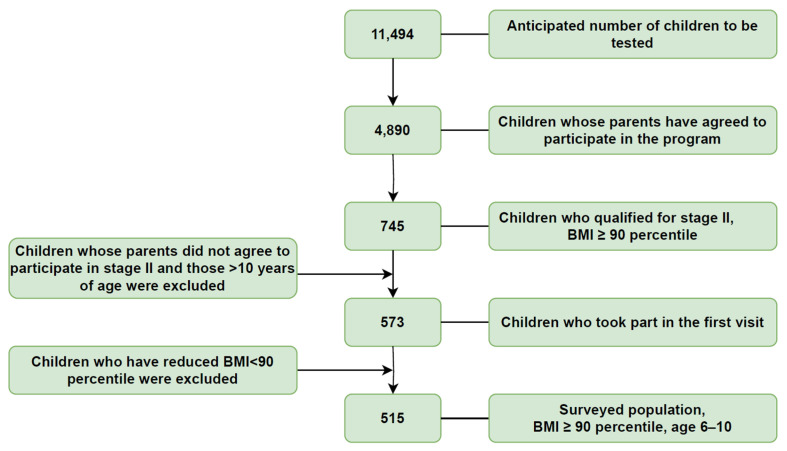
Schematic representation illustrating the criteria for inclusion in the study group.

The anticipated cohort for examination comprised 11,494 individuals, as obtained from the City of Szczecin Office. Fortuitously, 4890 parents in Szczecin provided their consent for participation, encompassing 2480 girls and 2410 boys. Within this cohort, 806 were diagnosed with overweight, constituting 16.9% of all participants, while 303 were identified with obesity, representing 6.4% of the total study populace. In aggregate, excessive body weight was prevalent in 23% of the participants. Due to the communal nature of the program, children from adjacent age groups were also included to prevent any sense of exclusion, resulting in a cohort that slightly deviated from the initially projected age range. Statistical analyses were primarily centered on data from 7-, 8-, and 9-year-olds, with a small subset of 6- and 10-year-olds. The second phase of the program enrolled 745 participants (408 girls and 337 boys) with a BMI ≥ 90th percentile, constituting 15% of the total participants. After excluding individuals who scheduled but did not attend their initial visit and those aged >10 years old, the cohort was narrowed down to 573 participants. Additionally, individuals who successfully reduced their BMI centile below the 90th percentile between the first and second stages of the program (58 participants) were excluded. Ultimately, the analysis focused on a cohort of 515 participants, comprising 1 child aged 6 years, 148 aged 7, 233 aged 8, 127 aged 9, and 6 aged 10 years. Schematic representation of projected and actual number of participants have been presented in the Figure 1.

Inclusion criteria for Stage I:

- Informed consent from a parent or legal guardian;
- Attendance in the second or third grade of elementary school (8 and 9-year-olds).

Inclusion criteria for Stage II of the program:

- Attendance in the second or third grade of elementary school (8 and 9-year-olds), BMI ≥ 90th percentile [14].

Exclusion criteria for Stages I and II:

- Absence of informed consent from a parent or legal guardian.

The program, owing to its societal nature, was tailored for students in all elementary schools in Szczecin, aiming to minimize exclusions and underscore inclusivity in its design.

### 2.3. Data Collection Procedure

#### 2.3.1. Anthropometric Measurements

Anthropometric parameters were consistently assessed using standardized procedures during both the initial screening phase and at each of the four intervention visits. Height measurements were obtained utilizing a Harpenden stadiometer with precision to 0.1 cm in the Frankfurt position. Body weight assessments employed a body composition analyzer with accuracy to 0.1 kg. Waist and hip circumferences were measured using a calibrated tape with precision to 0.1 cm.

#### 2.3.2. Blood Pressure

Preceding blood pressure measurements, participants observed a 5 min resting period in a tranquil environment, seated on a chair with back support, legs uncrossed, and feet flat on the floor. Physical activity was refrained for at least 30 min prior to examination. Blood pressure, measured three times, utilized an Omron 2 electronic blood pressure monitor with a appropriately sized cuff, placed directly on the patient’s arm without any intervening clothing. The arm was supported to align the cuff’s center with the right atrium level. Conversations were prohibited during measurement. Blood pressure evaluations were conducted consistently during the screening phase and at each of the four intervention visits [15].

#### 2.3.3. Body Composition Analysis

In the screening stage, body composition analysis employed a Jawon Medical X-Contact 350 body composition analyzer, while during the intervention stage, Jawon Medical IOI 353 utilized the electrical bioimpedance (BIA) method. Parameters determined included the percentage of body fat, fat mass, lean body mass, water mass, and muscle mass.

#### 2.3.4. Physical Fitness

Physical fitness was assessed using the Kasch Pulse Recovery Test Step, involving rhythmic stepping on a 30.5 cm platform for 3 min at a pace of 24 steps per minute, guided by a metronome. Heart rate was monitored with an electronic Polar analyzer during the 3 min exercise and 1 min and 5 s of rest. Only values recorded within one minute immediately after the test were analyzed. This method allowed an approximate assessment of physical fitness based on heart rate frequency, indicative of aerobic physical activity level, a fundamental aspect of a healthy lifestyle.

#### 2.3.5. The Doctor’s Office

The doctor’s office visit encompassed a comprehensive medical history gathering, coupled with a physical examination that included anthropometric measurements, blood pressure assessment, body composition analysis, and evaluation of physical fitness. Personalized recommendations were provided to the patient during each visit [16,17].

#### 2.3.6. The Physical Activity Room

An interview conducted by the physical activity specialist assessed the child’s engagement in physical education classes, screen time in front of electronic devices, and modes of transportation to and from school. Motor skills and physical fitness tests, encompassing abdominal and shoulder girdle strength, flexibility, standing long jump, and overall endurance, were administered. Recommendations were tailored to enhance core muscle strength, motor coordination, and endurance, with a focus on improving the quality of life through active recreation, outdoor leisure, and participation in organized group sports activities.

#### 2.3.7. The Dietitian’s Office

The specially designed nutritional interview was conducted by a dietitian during each visit. It included an assessment of the frequency and quality of breakfasts, consumption of sweets and sugary drinks, fruits, vegetables, dairy products, fish, nuts and seeds, legumes, fast food dishes, processed foods, the amount of water consumed, cooking techniques, and meal regularity. Based on the gathered interview data, dietary recommendations were formulated for every patient. Consultations were focused on nutritional education and monitoring of the progress of dietary behavior changes.

#### 2.3.8. The Psychologist’s Office

Psychological consultations involved a thorough understanding of the child, their family situation, coping mechanisms in stressful situations, their functioning in society, and an assessment of the dietary habits of the entire family. Additionally, patients and their families were supported and motivated to adhere to recommendations, achieve set goals and engage in active leisure time. Emphasis was placed on the importance of recognizing that the unit of change is the entire family, not just the child. New dietary rules were established collaboratively with the child, parents, and the specialist.

### 2.4. Data Analysis Methods

#### Biochemical Analyses

Venous blood was collected in the morning following an overnight fast and rest, utilizing vacuum tubes. Comprehensive analysis included the determination of glucose and insulin concentrations, lipid profile (total cholesterol, LDL and HDL cholesterol fractions, and triglycerides), as well as TSH, fT4, AST, and ALT assessments. Laboratory tests were conducted twice during the program to ensure a thorough evaluation. The following methods were employed:

- Glucose concentration was determined using an enzymatic method with hexokinase.
- Insulin concentration was determined using an electrochemiluminescence method (ECLIA).
- Total cholesterol concentration was determined using an enzymatic colorimetric method.
- HDL and LDL cholesterol concentrations were determined using a homogeneous colorimetric enzymatic method.
- Triglyceride concentration was determined using an enzymatic colorimetric method.
- TSH concentration was determined using an electrochemiluminescence method (ECLIA).
- fT4 concentration was determined using an electrochemiluminescence method (ECLIA).
- AST concentration was determined using a kinetic method. ALT concentration was determined using a kinetic method.

All analyses were conducted using the Cobas PRO c503 module by Roche Diagnostics. Reference values for glucose were 60–99 mg/dL. The reference range for insulin was 2.6–24.9 μIU/mL, and for liver enzymes: ALT 0–44 U/L, AST 0–50 U/L. Reference values for lipids were described in Table 1.

Based on the obtained results, the HOMA-IR index was calculated after both the first and fourth visits using the formula: WH=I0×G0/22.5, where

WH—HOMA-IR index,
I0—fasting insulinemia [μIU/mL], and
G0—fasting glycemia [mg/dL].

### 2.5. Statistical Analysis Methods

The statistical analysis employed Stata 11 (license number 30110532736) and MS Office Excel. Continuous variables underwent normality distribution checks utilizing the Kolmogorov–Smirnov test. These variables were summarized using mean values, standard deviations, standard errors, as well as minimum and maximum values.

As almost all variables adhered to normal distributions, and in instances where deviations existed, the discrepancy (Dmax) was minimal, and the sample size (N) was high. The comparison of continuous variables was executed through Student’s *t*-test or ANOVA. The variables were presented as the mean values, standard deviations, standard errors, and minimum and maximum values.

Results were elucidated using the correlation coefficient (r) and probability (*p*). Spearman’s rank correlation was applied to assess the correlation between qualitative and quantitative variables. The outcomes were conveyed through the correlation coefficient (r) and probability (*p*). In all conducted tests, statistically significant differences were defined as those where the probability (*p*) equaled or was less than 0.05. A significance level of *p* = 0.051–0.099 was designated as a trend bordering on statistical significance.

## 3. Results

The data were analyzed based on two cut-off points for the HOMA-IR index: 2.5 and 3.16. It is worth noting that despite a small percentage of children with abnormalities in glucose and/or insulin levels, a substantial proportion of children exhibited an elevated HOMA-IR index value.

### 3.1. Distribution of Biochemical Parameters in Examined Children after the First Visit

**Figure 2:**
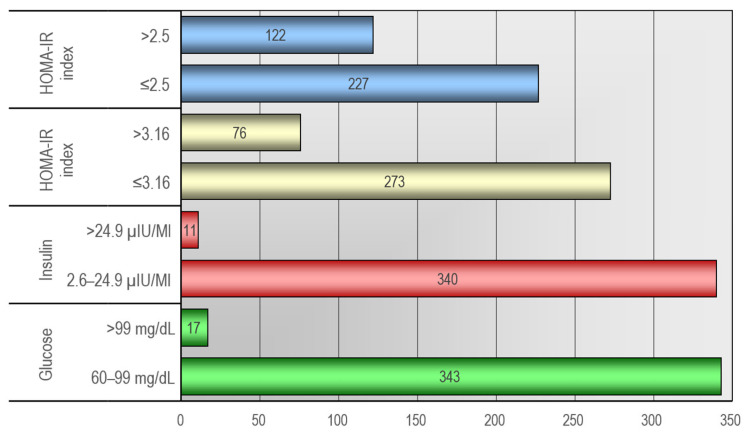
Distribution of carbohydrate metabolism parameters after the first visit.

In the analysis of biochemical studies, abnormalities associated with disorders in carbohydrate metabolism were evaluated. Elevated levels of the HOMA-IR index (>2.5) were observed in 122 children, while (>3.16) in 76. Increased insulin levels were noted in 11 patients, while elevated glucose levels were observed in 17 children. Detailed data illustrating disturbances in carbohydrate metabolism were presented in Figure 2.

**Figure 3:**
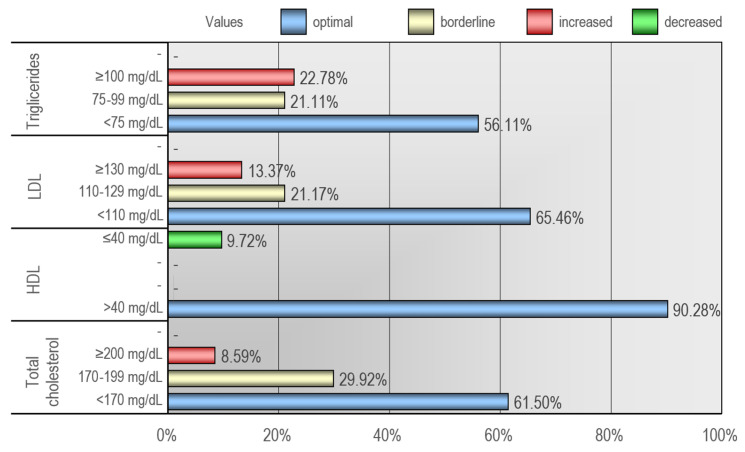
Distribution of lipid metabolism parameters after the first visit.

Elevated total cholesterol levels (≥200 mg/dL) were diagnosed in 8.6%, while borderline values (170–199 mg/dL) were observed in 29.9% of the children. Decreased HDL levels (<40 mg/dL) were diagnosed in 9.7% of the examined population. Elevated LDL levels (≥130 mg/dL) were diagnosed in 13.4%, and borderline levels (110–129 mg/dL) in 21.2% of the children. Elevated triglyceride levels were diagnosed (>100 mg/dL) in 22.8%, while borderline levels (75–99 mg/dL) were observed in 21.1% of the patients. Over half of the examined children with excess body weight exhibited at least one abnormality in their lipid profile. Detailed data illustrating disturbances in lipid metabolism were presented in Figure 3.

During visits 1 and 4, changes in the biochemical parameters of the studied population, among children who attended all specialist visits and underwent laboratory tests, were also assessed. For each biochemical parameter, the initial difference between these parameters between the 1st and 4th visits was calculated for every patient, and then the average was derived from the obtained data. It is worth mentioning that between the 1st and 4th visits, only a decrease in liver enzyme concentration was observed. A statistically significant increase in glucose concentration, LDL cholesterol, insulin, and the HOMA-IR index was observed. For the remaining laboratory tests, the differences were not statistically significant. Detailed data illustrating the variable changes in biochemical parameters have been gathered in Table 2A,B.

### 3.2. Comparison of Biochemical Parameters in the Fourth Group of Examined Children

A total of 195 children completed the fourth visit, while laboratory tests were conducted on 119 to 122 children (depending on the biochemical parameter analyzed). Therefore, in this analysis, the biochemical parameters checked after the first visit in children who completed all specialist visits were compared with parameters performed after the fourth visit in children who also completed all specialist visits, excluding children who did not undergo laboratory tests after the last visit. An increase in glucose concentration (*p* < 0.01, R = 0.19), LDL cholesterol fraction (*p* = 0.05, R = 0.11), insulin (*p* < 0.01, R = 0.19), and the HOMA-IR index (*p* < 0.01, R = 0.20) was observed, and these changes were statistically significant. A borderline statistically significant decrease in AST concentration was noted. No statistically significant changes were observed for the remaining parameters. Detailed data are provided in Table 3.

Analysis of biochemical parameters in examined children was conducted between those who underwent laboratory tests after their first visit and those who underwent tests after their fourth visit. When comparing the biochemical parameters of all children who had their first visit and conducted laboratory tests with those who completed all visits and underwent tests after the fourth visit, statistical significance was observed only for parameters related to carbohydrate metabolism. A significant increase was noted in the concentration of glucose (*p* < 0.01, R = 0.17), insulin (*p* < 0.01, R = 0.21), and the HOMA-IR index (*p* < 0.01, R = 0.22). No statistical significance was observed for the remaining biochemical parameters. Detailed data were presented in Table 4.

### 3.3. Analysis of Changes in Biochemical Parameters of the Examined Children during the Course of Four Specialized Visits

**Figure 4:**
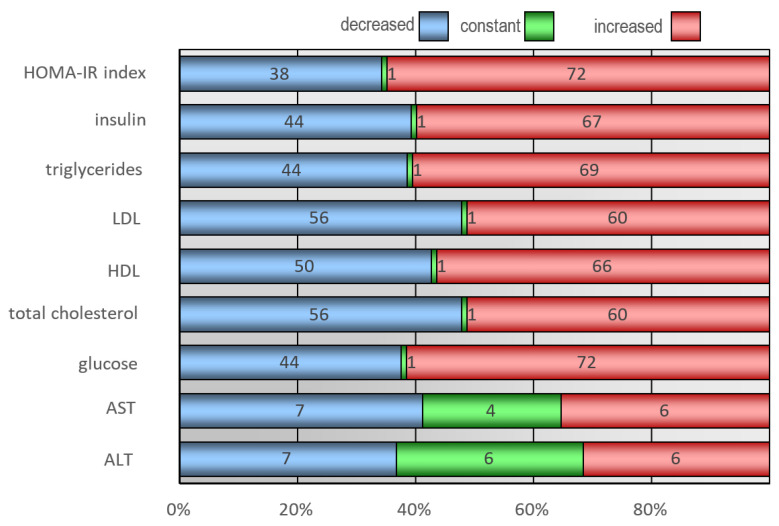
Changes in biochemical parameters of examined children during the course of four specialized visits.

In the analysis of changes in biochemical parameters during the year-long intervention program, an increase in the HOMA-IR index was observed in 72 children, elevated insulin levels in 67, heightened triglyceride levels in 69, increased LDL levels in 60, elevated total cholesterol levels in 60, elevated glucose levels in 72, and notably, increased HDL cholesterol levels in 66 children. Additionally, a reduction in parameters such as AST and ALT levels was observed in seven children for each marker. Changes in biochemical parameters during the course of four specialized visits have been presented in Figure 4.

The relationships between biochemical parameters and the gender of the examined children were also analyzed. A statistically significant correlation was found between glucose concentration and gender. Moreover, higher glucose concentrations were observed in boys during the 1st visit. This correlation was not observed during the 4th visit. The change in glucose concentration between the 1st and 4th visits was more significant in girls. Detailed data were provided in Table 5.

A relationship between insulin concentration and gender has been demonstrated. Girls exhibited higher insulin concentrations during the 4th visit. A similar relationship was not observed during the 1st visit. However, the change in insulin concentrations was also not statistically significant. Detailed data were presented in Table 6.

A correlation between the HOMA-IR index and gender was observed. A higher HOMA-IR index in girls during the 4th visit was noted, with statistical significance (*p* = 0.0160, R = 0.22). A similar correlation was not observed during the 1st visit. Furthermore, the change in the HOMA-IR index was also not statistically significant. Detailed data were provided in Table 7.

A relationship between ALT concentration and gender was observed. Higher ALT concentrations in boys during the 1st visit were statistically significant. A similar relationship was not observed in the 4th visit; however, it has been assessed in a small group of children. Detailed data are provided in Table 8.

A borderline statistically significant relationship was observed between HDL concentration and gender, with higher concentrations found in boys. Other relationships between anthropometric and biochemical factors were not statistically significant. No statistical significance was observed for AST concentrations and changes in AST within the studied group of children.

The association between the studied biochemical parameters and the degree of overweight was also analyzed. The analysis revealed a connection between the HDL cholesterol fraction in the overweight group of children compared to the obesity group. Higher HDL concentrations were observed in the overweight group. This relationship was statistically significant only during the 1st visit. A greater increase in HDL concentration between the 1st and 4th visits was observed in the overweight group, although this relationship was not statistically significant. Detailed data were presented in Table 9.

An association between triglyceride concentrations was observed in the overweight group of children compared to the obesity group. Higher triglyceride concentrations were observed in the obesity group. This relationship was statistically significant only during the 1st visit. A greater increase in triglyceride concentrations between the 1st and 4th visits was observed in the obesity group, although this relationship was not statistically significant. Detailed analysis was presented in Table 10.

A relationship between insulin concentration was demonstrated in the overweight group of children compared to the obesity group. Higher insulin concentrations were observed in the obesity group. This relationship was statistically significant only during the 1st visit. A greater increase in insulin concentration between the 1st and 4th visits was observed in the overweight group, although this relationship was not statistically significant. Detailed data were presented in Table 11.

Higher glucose concentrations were observed among children with obesity; however, no statistically significant relationships were identified. Detailed data were presented in Table 12.

The analysis revealed an association between the HOMA-IR index in the overweight group of children compared to the obesity group. Higher insulin concentrations were observed in the obesity group. This relationship was statistically significant only during the 1st visit. A greater increase in insulin concentration between the 1st and 4th visits was observed in the overweight group, although this relationship was not statistically significant. More data were displayed in Table 13.

In addition, no statistically significant relationships were observed between total cholesterol concentration and LDL cholesterol fraction.

### 3.4. Analysis of Anthropometric Parameters among Children Who Completed the Entire Intervention Program

Special attention in the analysis of anthropometric parameters was given to changes in the BMI index, especially BMI centile, and the BMI z-score, which are objective indicators. BMI values decreased until the third visit, with the most significant reduction between the first and second visits, while an increase was noted during the fourth visit. Statistical significance was established for BMI centile (*p* < 0.01) and BMI z-score (*p* < 0.01). Detailed data concerning BMI changes during entire intervention program were presented in Table 14.

### 3.5. Limitations of This Study

One notable limitation of this study was the relatively low percentage of children for whom parental consent was obtained to participate in the intervention segment. The 515 children included in this study represented 69% of the potential beneficiaries from the intervention program. However, the rate of consent aligns with patterns observed in other clinical research studies. Persistent low public awareness of the severe health implications of overweight and obesity, particularly in children, contributed to this limitation. Additionally, the study’s duration, confined to one year, may be insufficient for the realization of enduring effects in terms of weight loss. Extending the timeframe could potentially yield more substantial benefits for many children.

### 3.6. Future Studies

A promising avenue for future research involves exploring the impact of the intervention program on children with excess body weight across diverse age groups, specifically including cohorts aged 10, 12, and 16. Furthermore, a prospective follow-up study could be conducted to reassess the participants from the initial research described by the authors. This follow-up, scheduled for 3–4 years later, aims to evaluate the sustained effectiveness of the multidisciplinary interventions.

## 4. Discussion

The prevalence of excessive body weight in studied population exceeded 23%, with 16.9% patients classified as overweight and 6.4% as obese. The most pronounced effects of decreasing anthropometric parameters were evident up to the 3rd visit (within the initial 6 months of the program), diminishing thereafter due to the extended interval between the 3rd and 4th visits (6 months). Notably, a gradual decline in both BMI percentile and BMI z-score was observed during all visits [19]. The intervention program successfully achieved the intended effects inhibition of the increase in individual’s anthropometric parameters.

The primary objective of this study was an precise analysis of the biochemical parameters in the examined children, encompassing parameters related to carbohydrate and lipid metabolism, as well as the concentration of liver enzymes, serving as early indicators of liver steatosis. Initially, 4.7% of children exhibited abnormal fasting glycemia (>99 mg/dL), and 3.1% showed elevated insulin levels (>24.9 μIU/mL). Data were assessed based on two cut-off points for the HOMA-IR index: 2.5 and 3.16. A HOMA-IR index > 3.16 was identified in 21.8%, and >2.5 in 35.0% of the examined children. The relatively low percentage of children with abnormal glucose and insulin fasting levels may be attributed to the young age of the patients and the moderate degree of obesity. It is worth noting, that still significant proportion of children exhibited an elevated HOMA-IR index, indicating early disturbances in glucose homeostasis associated with excessive body weight. This underscores that impaired glucose levels or pre-diabetes manifest later than insulin resistance, which remains the dominant abnormality in glucose metabolism in children with obesity. It is plausible that fasting glucose levels within the norm range are maintained through a compensatory mechanism based on hyperinsulinemia, as reflected by HOMA-IR values. A simple criterion for assessing insulin resistance is the presence of fasting hyperinsulinemia. According to the 2012 OSCA recommendations (Obesity Services for Children), fasting insulin levels should be interpreted based on the degree of sexual maturation. For children in Tanner stages 1 and 2, fasting insulin levels should not exceed 15 mIU/L [20]. In our study, a more permissive cut-off point for insulin (24.9 mIU/L) was adopted in alignment with the laboratory standards used for evaluating biochemical parameters. The HOMA-IR (homeostasis model assessment index) is a relatively straightforward method for assessing insulin resistance [20,21]. Thus far, no specific threshold value has been established above which the HOMA IR index in the pediatric patient group indicates pathology. Some authors suggest that insulin resistance can be identified if the HOMA-IR is greater than 2 or 3 [2]. In adults, an HOMA-IR index > 2.5 indicates insulin resistance. However, Keskina et al., in their study, proposed a value greater than 3.16 [22]. Shashaj stated that values exceeding 1.68 in individuals with normal body weight signify a “non-physiological state” (3.42 in the group of children and adolescents) and may pose an elevated risk of cardiovascular diseases [23]. The 2012 OSCA guidelines recommended an HOMA-IR value > 4.5 as a cut-off point defining insulin resistance in children with excess body weight [20]. Due to considerable disparities in the interpretation of the HOMA-IR index, the decision was made to conduct the analysis based on two cut-off points. Physiological insulin resistance occurs during adolescence when insulin sensitivity decreases by 25–50%. This trend improves after maturation is completed. Unfortunately, insulin resistance in adolescence is often exacerbated by excess body weight [24]. Genetic, familial, and ethnic factors likely play a more substantial role in the development of insulin resistance than its severity or duration. This might elucidate why insulin resistance is not consistently diagnosed in children with advanced obesity. Nevertheless, the risk of developing insulin resistance escalates proportionally with visceral adipose tissue mass [2].

Some studies suggest that physical activity may exert a greater impact on improving insulin sensitivity than reducing the body mass index (BMI). Modifying dietary habits and increasing physical activity seem to be the most important interventions even if they are not followed by BMI decrease [25]. Regrettably, the current level of physical activity in children, especially those with excess body weight, is very low. A study conducted in 2018 in Szczecin, Poland, in which the main author participated, presented data evaluating physical fitness in a cohort of 3321 eight- and nine-year-old children. An analysis based on the Kasch Pulse Recovery Test Step revealed very poor physical fitness (test termination before completion due to excessively high heart rate HR > 180/min) in 151 children (4.5%), very poor physical fitness in 234 children (7%), poor physical fitness in 827 children (24.9%), satisfactory physical fitness in 961 children (29.2%), good in 650 (19.5%), very good in 428 children (12.8%), and excellent in 70 children (2.1%). Unfortunately, more than 1/3 of the tested population achieved an unsatisfactory physical fitness result [26]. The presented results of the study on carbohydrate metabolism disorders may further indicate the need for the application of different, higher norms for both insulin concentrations and insulin resistance indices during the adolescent period. These topics remain controversial and debatable [20,27,28]. It may also highlight the significant impact of excess body weight on metabolic disorders that appear relatively early, despite young age and despite improvements in anthropometric parameters.

Both parameters evaluating carbohydrate metabolism and those evaluating lipid metabolism could be specific indicators of increased risk of early disorders and complications related to excess body weight. Abnormalities in triglycerides compared to other lipids were distinctly noticeable, serving as the primary marker of lipid disorders associated with an improper diet and high consumption of simple sugars. Apart from elevated triglyceride levels, the most frequently observed disorder was elevated LDL cholesterol level. It is worth noting the significantly high percentage of borderline lipid levels in the studied population. Given the age of the studied children and the fact that they were not only obese but also overweight, such significant deviations in laboratory tests were not expected. Abnormalities in the studied biochemical parameters were found in over half of the examined population. In the analyzed group, lipid metabolism disorders were more prevalent than deviations in carbohydrate metabolism. It has also been demonstrated that the risk of developing lipid disorders is 2.8-fold higher in children with a BMI > 90th percentile compared to children with normal body weight. The prevalence of dyslipidemia in the population of obese children ranges from 27% to 43% [29]. Both HDL cholesterol and triglyceride concentrations are proportional to visceral adipose tissue mass, unlike LDL cholesterol levels. It is important to note that the unfavorable lipid profile and metabolic disturbances observed in children with excess body weight can persist into adulthood. Excessive dietary fat intake, as well as other poor dietary habits, can lead to dyslipidemia regardless of whether they affect weight gain. The most common dyslipidemia among obese children is hypo-HDL-cholesterolemia. The triglyceride-to-HDL cholesterol ratio (TG/HDL-C) is associated with increased BMI and cardiometabolic risk factors [2,30], and it also serves as a good marker for insulin resistance and diabetes [31]. A similar study focusing on the evaluation of biochemical parameters was conducted on a population of children with obesity in Portugal. The authors found decreased HDL cholesterol levels in 11% and elevated triglyceride levels in 13.4% of the studied population. No child showed elevated fasting blood glucose, but hyperinsulinemia was detected in 7.3% (>15 µUI/mL) and insulin resistance in 8.5% of the study group [32]. In our study, the percentage of children with decreased HDL cholesterol fraction was equal to 9.7%, while the percentage of children with elevated triglyceride levels was noticeably higher, equal to 21.2%. In a large Polish study conducted by Brzeziński between 2011 and 2017 as part of the “6-10-14 for Health” program, lipid, carbohydrate, liver enzyme, and TSH parameters were evaluated in children with overweight or obesity. This study revealed that nearly 40% of children with excess body weight were diagnosed with at least one abnormality in the lipid profile, with no statistically significant difference based on the gender of the children. In our study, this percentage was even higher, equal to 52.9%, and confirmed no gender-based significance. In Brzeziński’s study, the most frequently observed disorders were decreased HDL cholesterol levels in about 22% of children and increased LDL cholesterol levels in 15% of the studied population [12]. In our analysis, the most common lipid disorders were hypertriglyceridemia and elevated LDL cholesterol levels. Another study conducted by Nielsen et al., assessing lipid metabolism among Danish children, showed that lipid disorders occurred in 28% of the studied population and were significantly more common among girls [29]. A German study by Dathan-Stumpf et al. in 2016 revealed lipid disorders in 24.7% of children [33]. The frequent occurrence of lipid disorders in the pediatric population should serve as an important warning signal both at the individual and population levels. Importantly, screening tests used to diagnose lipid disorders should be applied from the earliest years. Changing dietary habits and increasing physical activity are crucial to improve and normalize lipid profile parameters. Juárez-López et al. evaluated 11–13-year-old Mexican children for complications related to excess body weight. They diagnosed hypolipoproteinemia in 69%, hypertriglyceridemia in 29%, fasting glucose abnormalities in 4%, and insulin resistance in 51% of the participants. They concluded that regardless of age and gender, a higher degree of insulin resistance is associated with a higher frequency of all components of the metabolic syndrome [34]. Deeb et al. assessed 216 children from Saudi Arabia with an average age of 10.58 ± 2.97 years. The majority of this group (93%) were children with obesity. They diagnosed dyslipidemia in 55.3%, from which 11.7% had elevated total cholesterol levels, 28.6% had elevated triglyceride levels, 32.7% had elevated LDL cholesterol levels, and 18.0% had low HDL cholesterol levels, emphasizing the significantly increased risk of developing complications associated with excess body weight [35].

Another important part of this study was the analysis of changes in biochemical parameters after a year of specialized interventions targeted at the children and their families. When comparing the differences in selected biochemical parameters between the 1st and 4th visits in the group of children who completed all specialized visits (Group 4) and underwent laboratory tests (n = 117), a statistically significant increase in glucose concentration by 2.15 ± 8.24 mg/dL, insulin by 2.72 ± 7.09 mg/dL, HOMA-IR by 0.68 ± 1.77, and LDL cholesterol fraction by 3.20 ± 17.0 mg/dL was observed. For other biochemical parameters, an increase in concentration was also noted but without statistical significance. It is worth noting that there was a decrease in the concentration of liver enzymes observed between the 1st and 4th visits and an increase in triglyceride levels, although without statistical significance, which would confirm the impact of dietary changes on triglyceride reduction. Biochemical parameters obtained during the first visit and the fourth visit from children who completed all specialized visits but excluding those who did not undergo laboratory tests after the last visit were compared. Similarly to the previous analysis, a statistically significant increase was observed in glucose concentration, LDL cholesterol fraction, insulin, and HOMA-IR. Additionally, a decrease in AST concentration was found at the borderline of statistical significance. What is more, increased ALT and glucose concentrations were observed in boys (during the first visit), while in the group of girls, there was an increase in carbohydrate metabolism parameters during the fourth visit. This effect could be attributed to the physiologically faster maturation and greater insulin resistance during this period in girls. Consequently, based on the obtained results of the analysis, it was questioned whether insulin concentration norms should be adjusted for gender in addition to age for children. Portuguese researchers observed significantly higher ALT concentrations in boys in their study group. Moreover, there was also a tendency for glucose, insulin, HOMA-IR index, and lipid concentrations (except HDL) to increase in boys, although without statistical significance [30]. The results align with the analysis conducted during this study. On the contrary, Holst-Schumacher et al., in their research, found higher insulin concentrations and HOMA-IR index accompanied by lower HDL cholesterol concentrations in girls [36]. Brzeziński emphasized significantly frequent elevated triglyceride in boys with excessive body weight [12]. In our study, no relationship was found between the gender of the examined children and lipid disorders. Interestingly, a study on cardiovascular risk conducted among young Finns revealed that after 21 years of observation, patients with a complex pattern of dyslipidemia that started in early childhood had significantly increased carotid intima-media thickness (cIMT) compared to the control group with normal lipid levels [37].

When analyzing individual biochemical parameters, the degree of obesity was taken into consideration. As expected, the higher the body weight, the more complications were found. Higher ALT concentrations were found in obese children compared to overweight, both during the first and fourth visits. In the group of overweight children, ALT levels decreased by 5 U/L between visits, while in the group of obese children, there was an increase of 1.42 U/L. These findings highlight that ALT can be a sensitive marker of liver cell dysfunction in the course of obesity and can increase rapidly with the degree of excessive body weight. However, this conclusion is based on a quite small-sized group.

During the first visit to the center, higher HDL concentrations were observed in the group of children with overweight, and higher HOMA IR, triglyceride, and insulin levels were noted in children with obesity. Higher values of the HOMA-IR index, indicating elevated levels of both glucose and insulin, were observed in obese children during the first visit and were borderline significant during the fourth visit. The observed relationships between HDL cholesterol, triglycerides, and insulin levels within the group of children with obesity were evident only during the first visits. This could result from the improvement in both anthropometric and biochemical parameters in the group of obese children after the completion of the intervention program. However, It is worth noting that these associations are characterized by a low correlation coefficient. Holst-Schumacher also described significantly higher levels of insulin, the HOMA-IR index, and triglycerides in the group of obese children, as well as lower levels of HDL within the same group of patients [36]. Moreover, research demonstrates that each component of the metabolic syndrome worsens with an increase in body weight. This association is independent of age, gender, and maturation. The prevalence of the metabolic syndrome significantly increases with growing insulin resistance as well [38].

Taking into account the debatable effectiveness of therapeutic interventions in the treatment of obesity and its complications, there has been a growing interest in preventive strategies. The evaluation of the results presented in this study raises questions about the effectiveness of preventive programs. Based on conducted research, it is evident that initial engagement in the program is high, but motivation tends to decrease over time. It is worth considering the frequency of specialized visits in both preventive programs and outpatient care for the patients with obesity. In this study, patients experienced deterioration of the results mostly between the 3rd and 4th visit, where the gap between visits was the longest, lasting 6 months. This suggests that a 3-month interval between checkpoints may be optimal to maintain motivation and achieve better results. The literature related to intervention programs is extensive, and research results are inconclusive. It is challenging to compare programs due to the diversity of interventions and the involvement of different patient environments. Moreover, limitations in interpretation arise from the lack of data on the long-term effects of the programs and their cost-effectiveness. Waters et al., in their study, confirmed the effectiveness of preventive interventions targeted at children with excess body weight aged 6 to 12 years [39]. The effectiveness of multidisciplinary intervention programs was confirmed by other authors evaluating a similar age group [40,41], however, there are also studies with less optimistic findings regarding the effectiveness of such programs [42]. It can be concluded that only integrated actions can increase awareness among patients and reverse the growing trend of obesity worldwide. For the greatest success, these actions must be coherent, interdisciplinary, and combine efforts from various environments. A priority area for future research should be preventing obesity from early childhood and shaping health-promoting behaviors at every stage of children’s development. Further research is essential to confirm the effectiveness of implemented interventions.

## 5. Conclusions

The one-year multidisciplinary intervention program successfully halted weight gain and enhanced anthropometric indicators, including BMI, BMI percentile, and BMI z-score, in the cohort of 8- and 9-year-olds with excess body weight. A notable proportion of children experiencing excess body weight exhibited disruptions in carbohydrate and lipid metabolism. Despite the positive changes in anthropometric parameters throughout the one-year intervention program, these metabolic disturbances endured post-intervention. The recurrent prevalence of carbohydrate and lipid disorders in the pediatric population should be recognized as a significant warning signal, both at the individual and population levels, particularly in the context of an elevated risk of developing complications related to obesity. There is a necessity for prolonged, multidisciplinary programs with regular follow-up assessments to avert the emergence of metabolic complications stemming from obesity and to mitigate excess body weight in children and adolescents.

## Figures and Tables

**Table 1:** Lipid Norms Based on AAP Guidelines [18].

Lipids	Age	Values (mg/dL)		
		Optimal	Borderline	High
Total cholesterol		<170	170–199	≥200
LDL		<110	110–129	≥130
HDL	<10 years	>40	30–40	
Triglycerides	<10 years	<75	75–99	≥100

**Table 2:** (A) Biochemical Variables of the Study Group. (B) Biochemical Variables of the Study Group.

**(A)**										
**Variable**		**Visit Number**		**n**	$\bar{x}$	**SD**		**Min.**		**Max.**
∆ALT		4		19	−0.95	6.30		−23.00		7.00
∆AST		4		17	−0.29	4.16		−13.00		8.00
∆Glucose		4		117	2.15	8.24		−27.20		25.90
∆Total Cholesterol		4		117	2.57	20.83		−42.10		74.00
∆HDL		4		117	0.41	8.78		−29.40		22.30
∆LDL		4		117	3.21	16.99		−39.10		50.80
∆Triglycerides		4		114	5.09	42.06		−136.10		136.10
∆Insulin		4		113	2.72	7.09		−15.30		31.30
∆ HOMA-IR		4		112	0.68	1.77		−3.97		7.60
**(B)**										
**Variable**	**n**		**1st Visit**		**4th Visit**		**Diference**		***p***	
			$\bar{x}$ **± SD**		$\bar{x}$ **± SD**		$\bar{x}$ **± SD**			
ALT	19		16.58 ± 5.66		15.63 ± 5.14		−0.95 ± 6.30		0.52	
AST	17		22.71 ± 5.29		22.41 ± 3.45		−0.29 ± 4.16		0.77	
Glucose	117		89.42 ± 6.36		91.57 ± 7.46		2.15 ± 8.24		0.01	
Total cholesterol	117		162.80 ± 29.50		165.40 ± 29.90		2.60 ± 20.80		0.18	
HDL	117		55.42 ± 13.42		55.82 ± 12.01		0.41 ± 8.78		0.62	
LDL	117		103.40 ± 26.80		106.60 ± 27.30		3.20 ± 17.00		0.04	
Triglycerides	114		82.34 ± 43.27		87.43 ± 44.31		5.09 ± 42.06		0.19	
Insulin	113		11.35 ± 6.45		14.08 ± 6.68		2.72 ± 7.09		<0.01	
HOMA-IR	112		2.55 ± 1.58		3.23 ± 1.65		0.68 ± 1.77		<0.01	

$\bar{x}$—mean; n—study group size; SD—standard deviation; min.—minimal value; max.—maximum value.

**Table 3:** Biochemical Parameters of the Examined Children in the Fourth Group.

Visit Number	n	$\bar{x}$	SD	Min.	Max.	*p*
Glucose						
1	184	89.24	5.82	71.20	104.00	<0.01
4	122	91.78	7.50	66.90	110.20	
LDL						
1	184	100.78	25.87	32.30	197.30	0.05
4	121	106.90	27.16	41.10	187.70	
Insulin						
1	180	11.51	6.61	1.50	44.10	<0.01
4	119	14.11	6.56	3.50	35.70	
HOMA-IR						
1	179	2.58	1.60	0.31	10.71	<0.01
4	119	3.24	1.61	0.73	8.45	

$\bar{x}$—mean; n—study group size; SD—standard deviation; min.—minimal value; max.—maximum value; *p*—probability.

**Table 4:** Biochemical Parameters after the First and Fourth Visits.

Visit Number	n	$\bar{x}$	SD	Min.	Max.	*p*
Glucose						
1	360	89.23	6.16	71.10	106.10	<0.01
4	122	91.78	7.50	66.90	110.20	
Insulin						
1	351	11.05	6.04	1.50	44.10	<0.01
4	119	14.11	6.56	3.50	35.70	
HOMA-IR						
1	349	2.47	1.45	0.31	10.71	<0.01
4	119	3.24	1.61	0.73	8.45	

$\bar{x}$—mean; n—study group size; SD—standard deviation; min.—minimal value; max.—maximum value; *p*—probability.

**Table 5:** Relationship between Glucose Concentration and Gender.

Variable	Visit Number	Girls			Boys			*p*
		n	$\bar{x}$	SD	n	$\bar{x}$	SD	
Glucose	1	196	88.19	6.12	164	90.48	5.99	<0.01
Glucose	4	68	92.42	7.48	54	90.97	7.51	0.29
∆Glucose	4	65	3.69	7.58	52	0.24	8.69	0.02

$\bar{x}$—mean; n—study group size; SD—standard deviation; *p*—probability; ∆—change.

**Table 6:** Relationship between Insulin Concentration and Gender.

Variable	Visit Number	Girls			Boys			*p*
		n	$\bar{x}$	SD	N	$\bar{x}$	SD	
Insulin	1	190	11.20	5.85	161	10.87	6.27	0.61
Insulin	4	67	15.39	6.58	52	12.47	6.21	0.02
∆Insulin	4	63	3.50	7.03	50	1.74	7.13	0.19

$\bar{x}$—mean; n—study group size; SD—standard deviation; *p*—probability; ∆—change.

**Table 7:** Relationship between the HOMA-IR Index and Gender.

Variable	Visit Number	Girls			Boys			*p*
		n	$\bar{x}$	SD	N	$\bar{x}$	SD	
HOMA-IR	1	189	2.47	1.4	160	2.48	1.52	0.98
HOMA-IR	4	67	3.56	1.63	52	2.84	1.52	0.02
∆HOMA-IR	4	62	0.91	1.75	50	0.4	1.77	0.13

$\bar{x}$—mean; n—study group size; SD—standard deviation; *p*—probability; ∆—change.

**Table 8:** Relationship between ALT Concentration and Gender.

Variable	Visit Number	Girls			Boys			*p*
		n	$\bar{x}$	SD	n	$\bar{x}$	SD	
ALT	1	196	16.83	6.28	163	18.95	10.75	0.02
ALT	4	16	16.19	5.42	6	15.67	5.05	0.84
∆ALT	4	14	−1.07	7.27	5	−0.6	2.61	0.89

$\bar{x}$—mean; n—study group size; SD—standard deviation; *p*—probability; ∆—change.

**Table 9:** Association between HDL Cholesterol Concentration in the Group of Overweight Children Compared to the Group of Obese Children.

Variable	Gender	Visit Number	Overweight			Obesity			*p*
			n	$\bar{x}$	SD	n	$\bar{x}$	SD	
HDL	both	1	173	57.68	14.89	187	54.05	12.78	0.01
HDL	both	4	59	56.81	12.79	62	54.64	10.99	0.32
∆HDL	both	4	59	1.18	9.45	58	−0.38	8.05	0.34

$\bar{x}$—mean; n—study group size; SD—standard deviation; *p*—probability; ∆—change.

**Table 10:** Association between Triglyceride Concentrations in the Overweight Group of Children Compared to the Obesity Group.

Variable	Gender	Visit Number	Overweight			Obesity			*p*
			n	$\bar{x}$	SD	n	$\bar{x}$	SD	
Tg	Both	1	173	74.1	36.6	187	85.1	47.5	0.01
Tg	Both	4	58	82.2	43.5	60	93.2	43.9	0.18
∆Tg	Both	4	58	3.4	41.6	56	6.87	42.83	0.66

$\bar{x}$—mean; n—study group size; SD—standard deviation; *p*—probability; ∆—change.

**Table 11:** Association between Insulin Concentration in the Overweight Group of Children Compared to the Obesity Group.

Variable	Gender	Visit Number	Overweight			Obesity			*p*
			n	$\bar{x}$	SD	n	$\bar{x}$	SD	
Insulin	Both	1	169	9.53	4.41	182	12.46	6.96	<0.01
Insulin	Both	4	57	13.09	6.62	62	15.05	6.42	0.1
∆Insulin	Both	4	57	3.01	6.15	56	2.43	7.98	0.67

$\bar{x}$—mean; n—study group size; SD—standard deviation; *p*—probability; ∆—change.

**Table 12:** Association between Glucose Concentration in the Overweight Group of Children Compared to the Obesity Group.

Variable	Gender	Visit Number	Overweight			Obesity			*p*
			n	$\bar{x}$	SD	n	$\bar{x}$	SD	
Glucose	Both	1	174	88.94	6.15	186	89.51	6.17	0.38
Glucose	Both	4	59	90.9	7.8	63	92.6	7.17	0.21
∆Glucose	Both	4	59	2.24	8.5	58	2.07	8.04	0.92

$\bar{x}$—mean; n—study group size; SD—standard deviation; *p*—probability; ∆—change.

**Table 13:** Association between HOMA-IR Index in the Overweight Group of Children Compared to the Obesity Group.

Variable	Gender	Visit Number	Overweight			Obesity			*p*
			n	$\bar{x}$	SD	n	$\bar{x}$	SD	
HOMA-IR	Both	1	168	2.12	1.05	181	2.8	1.69	<0.01
HOMA-IR	Both	4	57	2.99	1.64	62	3.48	1.56	0.09
∆HOMA-IR	Both	4	57	0.77	1.49	55	0.59	2.03	0.6

$\bar{x}$—mean; n—study group size; SD—standard deviation; *p*—probability; ∆—change.

**Table 14:** Anthropometric Parameters Calculated during Four Visits among Children Who Completed the Entire Intervention Program.

Visit Number	n	$\bar{x}$	SD	Min.	Max.	*p*
BMI						
1	195	22.56	2.31	18.70	33.80	0.06
2	193	22.32	2.38	18.83	34.85	
3	194	22.46	2.42	18.59	35.60	
4	195	22.95	2.58	18.01	37.34	
Centile BMI						
1	195	95.41	2.42	90.00	99.90	<0.01
2	193	94.45	2.88	85.00	99.90	
3	194	93.91	3.50	82.00	99.90	
4	195	93.37	4.57	74.00	99.90	
Z-score BMI						
1	195	1.89	0.28	1.33	2.74	<0.01
2	193	1.80	0.29	1.18	2.71	
3	194	1.76	0.32	1.07	2.63	
4	195	1.72	0.35	0.67	2.65	

$\bar{x}$—mean; n—study group size; SD—standard deviation; Min.—minimal value; Max.—maximal value; *p*—probability.

## Data Availability

The data presented in this study are available on request from the corresponding author. The data are not publicly available due to ethical restrictions.
